# Validating the Family History Scoring System application to identify Lynch syndrome families

**DOI:** 10.1186/1897-4287-9-S1-P26

**Published:** 2011-03-10

**Authors:** Ellen McGannon, Janet Shenal, Matthew A Strohhacker, Susan Fay, James M Church

**Affiliations:** 1 Taussig Cancer Institute, Cleveland Clinic, Cleveland, Ohio, USA; 2 Department of Colorectal Surgery, The Sandford R. Weiss, M.D. Center for Hereditary Colorectal Neoplasia, Digestive Disease Institute, Cleveland Clinic, Cleveland, Ohio, 44195, USA

## Background

The FHSS (a points-based scoring system) was previously used to identify families at high risk of an inherited colorectal cancer syndrome. AM-I (Amsterdam-I) families scored high (≤12), but some scored low (≥8) when scored from unaffected relative’s perspective [1]. The FHSS is being applied to Lynch families (MMR+) and familial colon cancer type X (Type X) to see if the scoring system would yield similar results.

## Method

Family members (1 proband, 2 siblings and 2 children of the sibling) in Lynch and Type X families identified from the Jagelman Registry database were scored according to the score sheet described in Table 1.

**Table 1:** 

	Points
***Proband***	
Affected	*3*

***First Degree Relatives(FDR)***	
Each FDR	3 each
1-FDR<50-yr, including proband)	3
1-FDR–same side family	Extra 3

***Second Degree relatives(SDR)***	
Each SDR	1 each
>1-SDR<50-yr	1
>1-SDR–same side family	Extra 1*

***Combination FDR and SDR***	
1-FDR and any SDR–same side family	2*

*only if maximum 3 points per family not exceeded

They were scored from perspective of an affected proband (AP) or unaffected proband (UP), affected (AS) or unaffected (US) sibling or a child of each sibling (Child).

## Results

91 probands (68 affected, 23 unaffected) in 48 AM-I, 14 Amsterdam-II (AM-II), 6 Amsterdam-Like (AM-Like), 10 Familial Colon Cancer (FCC), 6 no syndrome (syn) families and 7 Type X were scored. 197 relatives were scored (38 affected siblings, 23 Child AP/AS, 40 Child AP/US, 77 unaffected siblings, 9 Child UP/AS, 10 Child UP/US). AM-I and Type X median scores were higher than other syndromes (<12) and suggestive of HNPCC when scored from the perspective of the proband, sibling or child of AS. The median scores were lower in AM-II, FCC, AM-Like, and no syn families with fewer colon cancers (Table 2).

**Table 2:** Median Scores

	AP	AP’s AS	AP’s US	Child AP’s AS	Child AP’s US	UP	UP’s AS	UP’s US	Child UP’s AS	Child UP’s US
Total Syndromes	14	18	14	12	4	12	15	12	7	4
AM- I	17	18	17	12	5	12	16	12	9	4
Type X	14	17	14	14	4	12	15	12		
AM-II	12	12	12	7	2	13	13	15	9	5
FCC	12	13	9	10	2	10	10	8	8	10
AM-LIKE	12		12		4	4		4		1
No-Syn	6		6		2	6	6		6	

## Conclusion

Findings were similar to validation study conducted on AM-I families. The FHSS is a reliable tool to determine familial risk of colorectal cancer especially in AM-I families and depends on the perspective of person being scored. In small families, those with predominantly extra colonic cancers or high-risk polyps, or if affected relatives are more than a generation from an individual, the FHHS will not always identify high risk or Lynch families.
